# Supplementary material for: Reducing Sialylation Enhances Electrotaxis of Corneal Epithelial Cells
Source: Int J Mol Sci. 2023 Sep 20;24(18):14327. doi: 10.3390/ijms241814327 (PMC10531958; doi:10.3390/ijms241814327)
Supplement: Supplementary file 1 [file ijms-24-14327-s001.zip › ijms-2603645-supplementary.pdf]

Supplementary Materials:

This manuscript contains four supplementary videos and one supplementary figure:

Supplementary Video S1: hTCEpi cells under 100mV/mm with and without 3F-Neu5Ac (100μM).

Supplementary Video S2: hTCEpi cells under 30mV/mm with and without 3F-Neu5Ac (100μM).

Supplementary Video S3: hTCEpi cells under 100mV/mm with and without $Ac_4ManNAz$ (100μM).

Supplementary Video S4: hTCEpi cells under 100mV/mm with and without Kifunensine (20μg/ml).

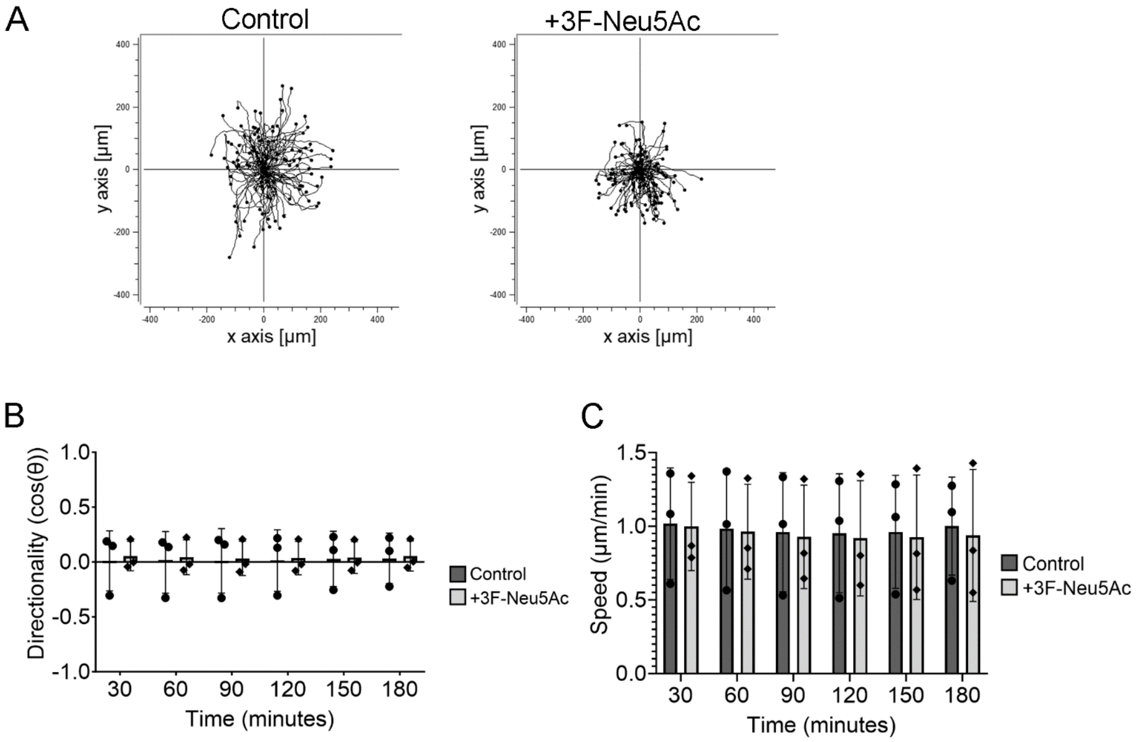

Supplementary Figure S1. 3F-Neu5Ac does not impact cell migration in the absence of an electric field. hTCEpi cells were seeded into electrotactic chambers and pretreated with 100 μM of 3F-Neu5Ac for 48 hours before electrical stimulation. (A) Representative trajectory plots of hTCEpi cells treated with or without 3F-Neu5Ac. (B) Quantification of directionality and speed (n=3). (C) Quantification of speed (n=3).
